# Regional insights on tobacco-related tweets: unveiling user opinions and usage patterns

**DOI:** 10.3389/fpubh.2024.1342460

**Published:** 2024-06-14

**Authors:** Consuelo Castillo-Toledo, Cesar I. Fernandez-Lazaro, Francisco J. Lara-Abelenda, Rosa M. Molina-Ruiz, Miguel Angel Ortega, Fernando Mora, Melchor Alvarez-Mon, Javier Quintero, Miguel Angel Alvarez-Mon

**Affiliations:** ^1^Department of Medicine and Medical Specialities, Faculty of Medicine and Health Sciences, University of Alcala, Alcala de Henares, Spain; ^2^Department of Psychiatry and Mental Health, Hospital Universitario Infanta Leonor, Madrid, Spain; ^3^Department of Preventive Medicine and Public Health, School of Medicine, University of Navarra, Pamplona, Spain; ^4^IdiSNA, Navarra Institute for Health Research, Pamplona, Spain; ^5^Departamento Teoria de la Señal y Comunicaciones y Sistemas Telemáticos y Computación, Escuela Tecnica Superior de Ingenieria de Telecomunicación, Universidad Rey Juan Carlos, Municipality of Fuenlabrada, Spain; ^6^Department of Psychiatry and Mental, Health San Carlos University Hospital (HCSC), Madrid, Spain; ^7^Research Biomedical Foundation of Clinico San Carlos Hospital (IDISCC), Madrid, Spain; ^8^Ramón y Cajal Institute of Sanitary Research (IRYCIS), Ramón y Cajal Hospital, Madrid, Spain; ^9^Department of Legal and Psychiatry, Complutense University, Madrid, Spain; ^10^Immune System Diseases-Rheumatology and Internal Medicine Service, CIBEREHD, University Hospital Príncipe de Asturias, Alcalá de Henares, Spain; ^11^CIBERSAM-ISCIII (Biomedical Research Networking Centre in Mental Health), Madrid, Spain

**Keywords:** tobacco, perception, geolocation, Twitter, Machine Learning, artificial intelligence

## Abstract

**Introduction:**

Tobacco consumption and its impact on health remain high worldwide. Additionally, it is a contentious issue generating significant controversy. Twitter has proven to be a useful platform for evaluating public health topics related to population health behaviors, and tobacco consumption.

**Objective:**

The objective of this study is to analyze the content of tweets related to tobacco. Moreover, geolocation data will be considered to understand regional differences.

**Methods:**

Tweets published between 2018 and 2022, in both English and Spanish, containing the keyword “tobacco,” were analyzed. A total of 56,926 tweets were obtained. The tweets were classified into different categories. 550 tweets were manually analyzed, and an automated and computerized classification was performed for the remaining and largest subset of tweets.

**Results:**

The analysis yielded 30,812 classifiable tweets. Healthcare professionals were the most frequent contributors to the topic (50.2%), with the most common theme being general information about the toxic effects of tobacco. 57.9% of the tweets discussed the harmful effects of tobacco on health, with fear being the predominant emotion. The largest number of tweets were located in America.

**Conclusions:**

Our study revealed a substantial number of tweets highlighting the health risks and negative perceptions of tobacco consumption. Africa showed the lowest percentage of tweets discussing the health risks associated with tobacco, coinciding with the continent having the least developed anti-tobacco policies. Healthcare professionals emerged as the most prominent users discussing the topic, which is encouraging as they play a crucial role in disseminating accurate and scientific health information.

## Introduction

The World Health Organization (WHO) estimates that there are 1.3 billion tobacco consumers globally, with Europe having the highest prevalence of tobacco use among adults (1). The health consequences of tobacco use are widely known. It is one of the leading preventable causes of premature death worldwide (2). Tobacco increases the risk of cardiovascular diseases (3), and it is a major cause of certain types of cancers such as lung and esophageal cancer (4). Additionally, it leads to lung diseases like COPD (Chronic Obstructive Pulmonary Disease) or chronic bronchitis (5).

The perception of tobacco in society has undergone a significant change in the last few decades. While tobacco was socially accepted and even idealized some years ago, currently, its consumption is widely regarded as harmful to health and carries a great social stigma (6). This change in tobacco perception has been driven by various factors, such as public health initiatives to raise awareness about the risks associated with tobacco use (7), scientific evidence linking smoking to numerous diseases (8), and the adoption of policies and regulations to restrict tobacco access and reduce consumption. The implementation of legislation to control tobacco consumption has had a significant impact on reducing tobacco use in many parts of the world (9). Various studies show that the implementation of anti-tobacco policies, such as smoking bans in public places, has led to a significant reduction in the number of smokers (10). Other measures promoted in 2005 by the WHO, in the Framework Convention on Tobacco Control, included the implementation of warning labels on tobacco packages (11). In this regard, some studies have been conducted, demonstrating their effectiveness, especially among non-smokers, and their ability to evoke emotions of aversion toward tobacco (12). One study measured the impact of various measures implemented for tobacco control, and found that among the most effective were the implementation of smoke-free public spaces (13). For example, in Spain, after implementing various measures to control tobacco consumption, tobacco sales decreased by 51% between 2005 and 2019 (14).

Traditional research methods such as surveys, interviews, cohort studies or naturalistic approaches have been the main and most used methods to investigate patients' and healthcare providers' experiences. However, these methods have several limitations. For example, they are subject to social desirability and recall bias or the inability to gather information in real-time (15–17).

As an alternative and innovative approach, social media platforms are increasingly being used by researchers for public health surveillance (18), as they provide a useful tool to capture more candid and natural opinions from users (19), which may not be obtained in more formal settings like medical consultations (20). Furthermore, this new research methodology allows health care professionals to listen to those patients who might be reluctant to participate in surveys and questionnaires through traditional methods (21).

Platforms like X (previously known as Twitter) host real-time, spontaneous discussions, offering an authentic window into the nuances of patient attitudes toward a certain topic, which are often missed by time-lagged traditional research methods. In a systematic review conducted to analyze the advantages and disadvantages of using Twitter in public health research, it was found that it is a valuable tool for identifying social concerns and information needs on a specific topic, but as a source of information, greater involvement of healthcare professionals is needed to improve the quality and accuracy of the messages (22).

Indeed, Twitter has proven to be a useful platform for evaluating public health topics related to tobacco legislation (23), population health behaviors (24), and tobacco consumption (25).

However, despite efforts to control tobacco use, the consumption of tobacco-related products remains high, and policies aimed at eradicating tobacco use continue to generate significant controversy.

In this study, we have formulated two hypotheses. Firstly, we hypothesize that the societal consideration of tobacco regarding personal experiences and health consequences has changed among the population due to anti-tobacco policies implemented in recent decades. Secondly, we assume that it is possible to identify geographical differences in opinions and concerns regarding tobacco consumption, which provide insights into user attitudes in different parts of the world.

Therefore, this article aims to examine whether there has been a shift in society's perception of tobacco and what prevailing opinions exist regarding anti-tobacco policies. To achieve this, we have collected tweets published on the topic between January 1, 2018, and April 30, 2022, analyzing the content, the type of user posting on Twitter about the topic under study, perceptions of its health effects, and personal experiences with consumption, taking into account the geolocation of the tweets to explore differences among different continents.

## Methods

### Search and data collection strategy on Twitter

This mixed-method, quantitative and qualitative analysis focused on the content of tweets related to tobacco published on the social media platform Twitter. We will explain the qualitative analysis in greater detail in the section titled “Identification of Thematic Categories and Creation of a Codebook,” while the more quantitative analyses are outlined in the section titled “Machine Learning Classification.” The combination of quantitative and qualitative methods allowed us to gain a broader view of the issue (26), and it also offers a more comprehensive and in-depth approach to understanding perceptions and attitudes related to tobacco on social media.

We included tweets that met the following inclusion criteria: (1) Public tweets; (2) Containing the word “Tabaco” or “tobacco” in the tweet text; (3) Published between January 1, 2018, and April 30, 2022; (4) Written in English or Spanish; (5) Receiving at least 10 retweets. These inclusion criteria were chosen to capture a broad and representative discussion on social media about the topic. We decided to collect data published over the past few years to cover a broad time span. Most studies of this nature are limited to days, weeks, or months. Very few studies analyze social media posts (or other internet spaces) over multiple years.

The tool used for collecting tweets is Tweet Binder, which has been widely used in previous research and provides access to 100% of public tweets (27, 28). Besides the tweet text, this tool provides the count of retweets and likes for each tweet, as well as the date of publication, a link to the tweet in its context, user description, and geolocation data. The number of retweets and likes received by each tweet serves as an indicator of the interest generated by the corresponding content among users (29).

### Identification of thematic categories and creation of a codebook

Using the previously mentioned search criteria, we collected 17,072 tweets in Spanish and 39,854 tweets in English. Subsequently, we conducted a content analysis using a mixed inductive-deductive approach to develop a codebook for classifying the tweets based on key thematic categories. A manual classification was performed on a small subset of tweets (*n* = 100) by two members of the research team. We created a codebook based on our research questions, our previous experience in analyzing tweets, and what we determined to be the most common themes. After discussing discrepancies and reaching a consensus on the codebook, an additional 450 tweets were then analyzed manually. This process also provided a larger sample for training the Machine Learning model. Finally, the remaining and larger subset of tweets (*n* = 56,926) was classified through an automated and computerized process.

The tweets were classified as classifiable or non-classifiable. A tweet was considered non-classifiable if its content was not related to the objectives of this study, if the content was insufficient to contain relevant information, or if it was written in a way that made its meaning uncertain. For each of the classifiable tweets, the content was analyzed based on the following themes: (1) Tweet topic; (2) Effect assessment; (3) Personal experience with tobacco; (4) Type of consumption. Finally, the users were classified into four categories: (1) General Twitter users; (2) Media; (3) Celebrity; and (4) Health professionals. The classification criteria and examples of tweets are shown in Table 1.

**Table 1:** Category, definitions and examples of classification.

**Category**	**Examples**
Effect assessment *(Whether consumption is perceived as beneficial or a health risk.)* • Health benefit • Harmful for health	I took a chance and switched to vaping not knowing that I'm minimizing my chances of harm from tobacco products and I'm winning. Life is lighter and fresher with vaping. I'm so happy with my vapes. • Marijuana can cause memory loss. Cocaine can causes brain damage. Tramadol can cause delusions. Skunk can cause lung damage. Colorado can cause psychosis. Rohypnol can cause amnesia. Tobacco can cause cancers. Ecstasy can cause seizures. Codeine can cause coma.
Topic • Claim *[Refers to both police/social/political complaint/claim (for or against)]* • General information *(Refers to when talking about more scientific issues)*. • Sale/advertising *(Tobacco is advertised)*. • Testimonials *(Regarding consumption, experience, more from the opinion of drug users or families/friends)*. • Trivialization. *(Minimization of the consequences of consumption, stigmatization, humorous tweets)*	• The highly profitable tobacco industry should pay more into cessation services and to improve people's health and wealth. That's why I support a “Polluter Pays” levy. If the Gov't is serious about its SmokeFree 2030 aims it should act now. My Westminster Hall speech • Varenicline is a drug used in smoking cessation. Varenicline is a partial agonist of the nicotinic receptor reducing both withdrawal symptoms and the rewarding effects of smoking by preventing binding of tobacco-derived nicotine to receptors. • Good news for tobacco firms—their vaping products will be paid for by the NHS in England—that's us • I took a chance and switched to vaping not knowing that I'm minimizing my chances of harm from tobacco products and I'm winning. Life is lighter and fresher with vaping. I'm so happy with my vapes • #GodMorningThursdayTobacco has originated from cow's blood. It is a sin to smoke tobacco
Personal experience with tobacco. *(Individual experience with tobacco, whether through family members, friends, or personal use.)*	• I took a chance and switched to vaping not knowing that I'm minimizing my chances of harm from tobacco products and I'm winning. Life is lighter and fresher with vaping. I'm so happy with my vapes
Consumption type. *(If talking about using tobacco frequently or only occasionally or binge)*	• Chronic smokers who switched from tobacco cigarettes to e-cigarette vapes in a large randomized control trial saw a significant improvement in markers of heart health after just a month.
User type *(Refers to the person sharing the tweet.)* • Health professionals. *(Healthcare professionals and healthcare institutions are included.)* • Undetermined. *(General population or it is not possible to identify)* • Media. • Celebrity. *(Any famous person; singers, actors, politicians, influencers...)*.	• Varenicline is a drug used in smoking cessation. Varenicline is a partial agonist of the nicotinic receptor reducing both withdrawal symptoms and the rewarding effects of smoking by preventing binding of tobacco-derived nicotine to receptors. • So many elderly people wake up to smoke each morning. They need Hukkah/Tobacco as soon as they get up—they don't realize they are killing themselves each day. Intoxication of any kind is dangerous to human life &amp; should be abandoned right away! #GodMorningMonday#mondaythoughts • A longtime Russian business associate of American tobacco giant Philip Morris International has been sanctioned in Europe for aiding Russia's invasion of Ukraine according to a @Reuters review of business registries and sanctions lists • We want to hear your perspectives on whether the #Tobacco and #Vaping Act is making progress toward achieving its vaping objectives. The consultation closes on April 27th 2022.

Usernames and personal names were removed.

### Ethical aspects

This study has been conducted following the ethical research principles outlined in the Declaration of Helsinki (seventh revision, 2013) and has received approval from the ethics committee of the University of Alcalá. Furthermore, it did not directly involve human subjects nor include any interventions. Only publicly available tweets were used (subject to universal access through the internet in accordance with the Terms of Service that all users accept on Twitter). In any case, we have taken care not to directly disclose any usernames in this work and have avoided citing information that could identify specific individuals.

### Machine learning classification

Technological advances in recent years have allowed the development of multiple emerging scientific disciplines, among them artificial intelligence (AI). AI refers to algorithms that seek to imitate human cognitive function through machines in order to perform data processing and analysis tasks (30). Within AI we can find several branches and one of them is Machine Learning, ML whose objective is to create computational models that extract knowledge from data with a reasonable capacity for generalization. Finally, within ML you can find Deep Learning (DL) (31). DL uses models called neural networks, which are AI methods inspired by human brain neurons whose function is to process information (31). Neural networks have multiple applications ranging from weather prediction (32), through coronavirus detection (33) or the detection of objects in images (34). One of the fields where neural networks are widely used is in Natural Language Processing (NLP). In NLP, networks are used on text to detect emotions, summarize documents, or even extract key ideas (35). In this project, a network pretrained on 850 million English tweets called BERTWEET (36) has been used to classify tobacco-related tweets into different categories.

A preprocessing of the database was necessary before the application of the BERTWEET network. All non-English tweets were translated into English since the network is trained only in English tweets. Thus, Google Translator was used for the translation of the non-English tweets. Then, the tweets were normalized by removing special characters such as @ or #, separating the negative tenses (don't into do not) and removing repeated characters. Finally, BERTWEET is a network that is not trained to classify into the categories we need, so it was necessary to retrain it in a process called fine-tuning. The manually classified tweets were randomly separated into two subsets, one for training composed by 80% of the tweets and another for testing composed of 20% of the tweets. The train subset was used to apply the fine-tunning to the network, whereas the test subset was used to validate that the fine-tunned version of the BERTWEET has a correct performance in our database. The methodology was adopted previously, and it seemed to work well in another context (37). Finally, we used the fine-tunned BERTWEET model (trained to apply our classification) to categorize the tweets that had not been classified by hand.

Furthermore, we have analyzed the emotions of the tweets, by applying a pretrained neural network called emotion-English-distilroberta-base (38). This neural network does not need a fine-tunning phase since it was used for the same purpose as it was previously trained. Emotion-English-distilroberta-base is a network capable of detecting Ekman's 6 basic emotions (39) plus the neutral emotion, making a total of 7. This network has already been used previously in other research studies (40). The model was applied to the 56,926 tweets, previously translated into English, and normalized.

### Statistical analysis

Descriptive statistics included frequency, proportions, and ratios to summarize number of tweets, likes and retweets. The ratio of like per tweet was calculated by dividing the number of likes by the number of tweets, while the ratio of retweet per tweet was calculated by dividing the number of retweets by the number of tweets. All statistical analyses were performed with SPSS version 16.0 software (SPSS Inc, Chicago, IL).

## Results

### The harmful effects of tobacco generate significant interest among Twitter users

According to the codebook, a total of 30,812 classifiable tweets were obtained. Among these, more than half, 16,086 (52.2%) tweets, were posted by healthcare professionals, although they had a lower impact in terms of retweets and likes (Table 2). Approximately 57.9% (17,850) of Twitter users expressed their opinions about the harmful effects of tobacco consumption, which is six times more than the tweets discussing the benefits of tobacco use (Table 2).

**Table 2:** Descriptive characteristics of the tweets considered classifiable in the content analysis.

	**Tweets**		**Number likes/number Tweets**	**Number retweets/number Tweets**
	**n**	**%**	**-**	**-**
Overall	30,812	100	–	–
**Effect assessment**				
No mention	10,139	32.9	151.5	52.0
Health benefit	2,823	9.2	183.8	50.9
Harmful for health	17,850	57.9	200.1	75.6
**User type**				
Health professionals	16,086	52.2	143.6	55.5
Undetermined	1,792	4.6	550.2	121.8
Media	3,562	11.6	157.0	57.9
Celebrity	9,372	30.4	189.0	75.1
**Topic**				
Claim	5,700	18.5	149.6	56.7
General information	13,706	44.5	132.3	56.6
Sale/advertising	1,532	5.0	105.5	46.8
Testimonials	8,310	27.0	305.2	79.7
Trivialization	1,564	5.1	168.4	120.0
**Personal experience with tobacco**				
No mention	7,750	25.2	120.4	46.4
Positive	3,711	12.0	248.6	59.5
Negative	19,351	62.8	194.9	74.4
**Consumption type**				
No mention	27,218	88.3	184.6	66.2
Frequent consumption	3,551	11.5	164.4	60.3
Occasional/binge consumption	43	0.1	406.3	123.8

Regarding the topic of discussion, the most common theme, with 13,706 tweets (44.5%), was related to general information about tobacco. This includes consequences, health implications, preventive measures, and awareness campaigns (Table 2). As for personal experiences with tobacco consumption, 62.8% of the tweets (19,351) identify them as negative (Table 2). Regarding the type of tobacco consumption, approximately 11.6% of the tweets addressed this aspect, with 11.5% specifically discussing frequent consumption (Table 2).

**Figure 1:**
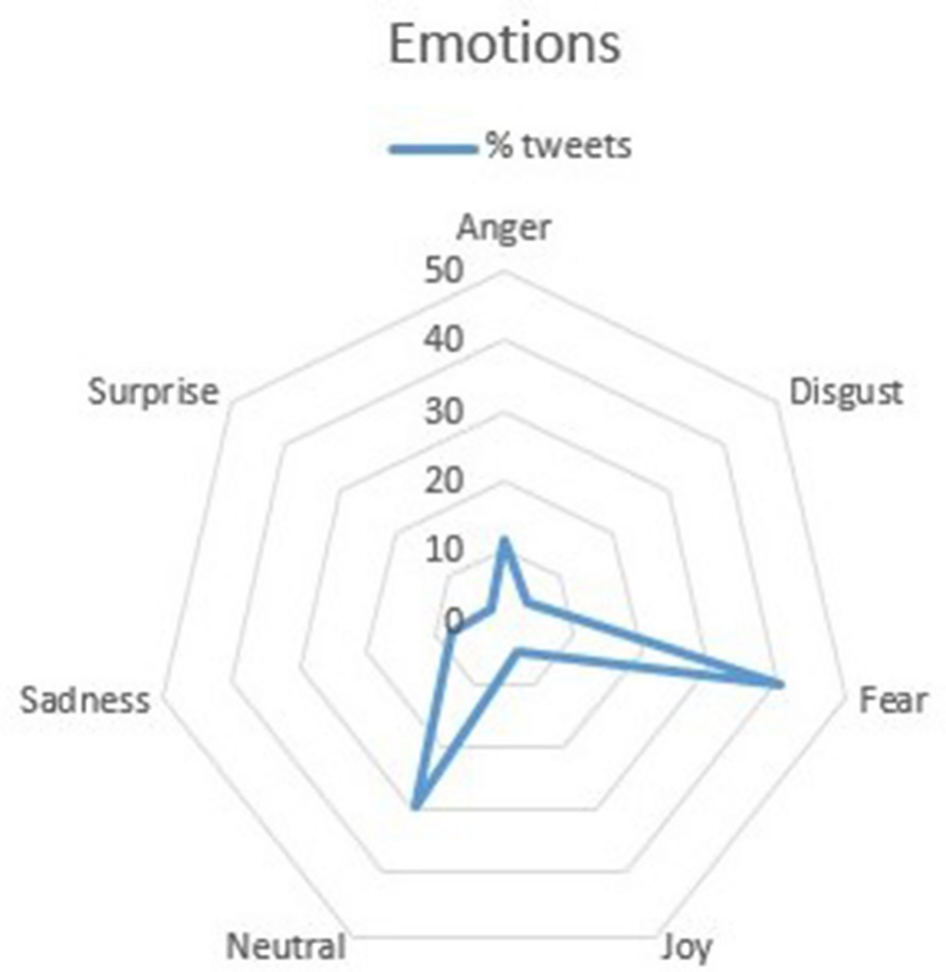
Emotions analysis. % tweets of each emotion.

In the emotion extraction analysis, as depicted in Figure 1, fear is the most frequent emotion, present in 40.4% of the tweets. However, tweets expressing disgust have the highest number of likes and retweets.

### Content analysis by continents

Out of the 22,647 geolocated tweets, the continent with the highest number of tweets is America, with 10,516 tweets, representing 34.1% of the total results. When conducting a content analysis by continents (Table 3), there is a higher number of tweets posted by healthcare professionals. Additionally, the most frequent theme is also “General information about tobacco,” except for the African continent, where there is a higher number of tweets expressing social or political criticism, representing 36.5% of the tweets. Moreover, in Africa, media outlets have a greater presence, accounting for 25.9% of the published tweets. In terms of user type, Asia has a higher percentage of tweets posted by public figures compared to other continents, representing 44.5%.

**Table 3:** Number of tweets by continent and category of the codebook.

	**America**	**Europe**	**Africa**	**Asia**	**Oceania**
	***n*** **(%)**	***n*** **(%)**	***n*** **(%)**	***n*** **(%)**	***n*** **(%)**
*Overall*	10,516 (34.1)	6,173 (20.03)	2,249 (7.29)	3,719 (12.07)	866 (2.81)
**Effect assessment**					
No mention	3,970 (37.8)	1,755 (28.4)	741 (53.6)	766 (20.7)	297 (34.3)
Health benefit	965 (9.2)	678 (11.0)	67 (4.8)	191 (5.2)	114 (13.2)
Harmful for health	5,581 (53.1)	3,740 (60.6)	575 (41.6)	2,752 (74.2)	455 (52.5)
**User type**					
Health professionals	5,266 (50.1)	3,982 (64.5)	543 (39.3)	1,754 (47.3)	483 (55.8)
Undetermined	511 (4.9)	474 (7.7)	53 (3.8)	40 (1.1)	15 (1.7)
Media	1,332 (12.7)	611 (9.9)	358 (25.9)	264 (7.1)	52 (6.0)
Celebrity	3,407 (32.4)	1,106 (17.9)	429 (31.0)	1,651 (44.5)	316 (36.5)
**Topic**					
Claim	2,253 (21.4)	846 (13.7)	505 (36.5)	406 (11.0)	181 (20.9)
General information	4,896 (46.6)	3,249 (52.6)	498 (36.0)	1,359 (36.6)	368 (42.5)
Sale/advertising	567 (5.4)	286 (4.6)	74 (5.4)	123 (3.3)	56 (6.5)
Testimonials	2,686 (25.5)	1,683 (27.3)	292 (21.1)	983 (26.5)	245 (28.3)
Trivialization	114 (1.1)	109 (1.8)	14 (1.0)	838 (22.6)	16 (1.9)
**Sentiment related to consumption**					
No mention	2,951 (28.1)	1,576 (25.5)	576 (41.7)	652 (17.6)	180 (20.8)
Positive	1,365 (13.0)	707 (11.5)	103 (7.5)	305 (8.2)	108 (12.5)
Negative	6,200 (59.0)	3,890 (63.0)	704 (50.9)	2,752 (74.2)	578 (66.7)
**Consumption type**					
No mention	9,337 (88.8)	5,147 (83.4)	1,275 (92.2)	3,503 (94.4)	797 (92.0)
Frequent consumption	1,164 (11.1)	1,018 (16.5)	105 (7.6)	206 (5.6)	68 (7.9)
Occasional/binge consumption	15 (0.1)	8 (0.1)	3 (0.2)	0	1 (0.1)

Regarding the effect assessment, Asia has the highest percentage of tweets discussing the harmful effects of tobacco, with 74.2% and Africa with the lowest percentage in this aspect. Additionally, in this continent, there is a higher number of tweets with negative sentiment related to tobacco consumption, representing 74.2% of the tweets. Lastly, in Europe, there is a higher frequency of content related to frequent tobacco consumption, with a total of 1,018 tweets (16.5%).

**Figure 2:**
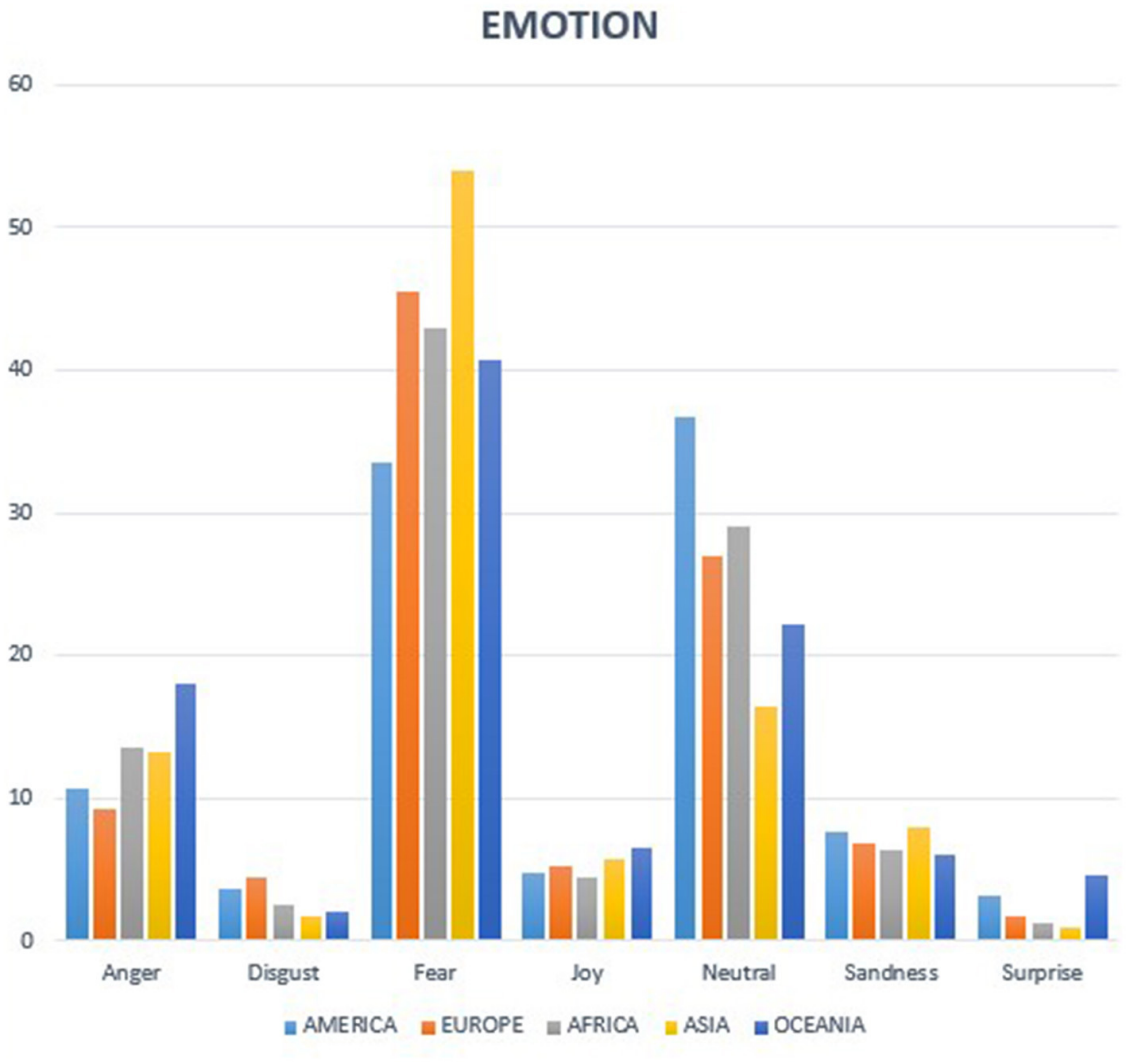
Emotion analysis by continents.

Regarding the emotion extraction analysis (Figure 2), fear is the predominant emotion in all continents, similar to the overall analysis. Nonetheless, there are some differences in the distribution of emotions across continents, with the Asian continent standing out. In the Asian continent, more than half of the tweets (54%) express fear, making it the continent with the highest percentage of fear-related tweets. On the other hand, in the continent of America, the trend is different, as 36.7% of the tweets are neutral.

## Discussion

In this work, we have found that there are six times more tweets about the harmful effects of tobacco on health than about its benefits, which is a very encouraging finding concerning the social awareness of users. This trend aligns with previous studies showing an increasing focus on the health risks of tobacco consumption, which could suggest that the implemented policies in recent years have been effective. In Europe, the proportion of smokers continues to decrease, with the majority of countries experiencing a decline from 32% to 26% since 2006 (41). Additionally, the most prevalent emotion in the tweets is fear, accounting for 40.4% of the posts, which is a positive result as this emotional response can prompt individuals to quit smoking (42). This emotional reaction is often associated with the population's knowledge about the physical health risks of tobacco consumption (43).

It is concerning that there is still a 9.2% of users who consider smoking to be beneficial. New forms of tobacco, such as e-cigarettes and vaping, may play a crucial role in this aspect, as they are sometimes perceived as less harmful by some individuals (44). Twitter conversations often promote the use of e-cigarettes and vaping as socially acceptable practices, discrediting scientific evidence of health risks, and advocating for minimal regulation of these products (45). This poses a new challenge in debunking the misconception that these new forms of tobacco are harmless and reducing their consumption. Some measures have already been implemented to tackle this new epidemic (46). For example, in the United States, the Federal T21 Law was passed in late 2019, raising the minimum age for the sale of tobacco products to 21 years (47, 48).

Interestingly, tweets regarding the supposed health benefits of smoking have achieved the same level of engagement as tweets about the health harms of smoking. Looking at the engagement generated by different types of users, it is surprising that undetermined users, despite being a minority, have clearly generated more engagement than the rest, which is contrary to what has been reported in previous studies that used a similar methodology to study other health issues. For example, in a study that examined common opinions and beliefs about electroconvulsive therapy, the users who generated the most engagement were healthcare professionals (37). In another study focusing on opioid-related posts on Twitter, it was also found that healthcare professionals and institutions were the users who generated the most engagement (49). Similarly, a recent study on societal views regarding cocaine also found that tweets published by healthcare professionals generated the most engagement (50). Therefore, the fact that in our study both tweets discussing the supposed benefits of smoking and those discussing the health harms of smoking have achieved very similar levels of engagement may be due to the presence of a few accounts classified as undetermined, which have many followers and are promoting the supposed benefits of tobacco. This assumption cannot be dismissed since previous studies have demonstrated that the tobacco industry uses social media to position itself and promote its sales (51, 52). Furthermore, it is well known that in other sectors, industries promote their products through social media. Indeed, food, beverage, and snack companies promote their brands on social media platforms, and use posts to advertise unhealthy products (53, 54). In the case of tobacco, this is particularly dangerous because exposure to such content on social media increases the likelihood that a young person will start smoking (40, 55).

The significant presence of healthcare professionals on Twitter, with 50.1% of the posts coming from this group, is a very positive finding. The use of social media by healthcare professionals facilitates the dissemination of health-related information and fosters bidirectional communication with users (56). Due to the higher number of tweets published by healthcare professionals, it is logical that the most recurring theme is general information about tobacco, scientific topics, health effects, and preventive measures. In light of these results, Twitter could be a useful platform for disseminating messages by healthcare professionals and institutions for tobacco prevention.

Regarding frequent tobacco consumption, only 11% of the analyzed tweets discuss this aspect. This finding does not align with more traditional data collection methods; according to the World Health Organization (WHO), 22.3% of the global population were smokers in 2020 (1). The discrepancy between official surveys and this study's analysis may be attributed to the exclusion of the keyword “vaping,” as the population might not consider vaping the same as tobacco consumption. Twitter discourse tends to view vaping as not posing health risks (43, 57), and the population is exposed to advertising related to e-cigarettes as a smoking cessation aid (58). The dissemination of preventive campaigns against the minimization of the risk of new tobacco products would be interesting. Social media could be a useful tool, as they are more cost-effective and currently have a larger audience than traditional media outlets (television, press) (59).

Another important group of tobacco influencers on Twitter are celebrities, accounting for 30.2% of the sample. We should not overlook this data as they often hold prominent positions on social media and have a large number of followers, which means their opinions reach a significant audience. A study on opinion leaders and general users on Twitter and their behavior and attitude toward tobacco products found that opinion leaders reported the highest prevalence of consumption. Therefore, they may be negatively influencing the rest of the population, especially the youth (60). Also, in another study, it was found that social influence on Twitter was primarily related to popularity (61). Messages shared by celebrities can influence public opinion and online discourse of other users (62).

A novel aspect of our work is the geolocation of tweets. It is notable that America is the continent with the highest number of geolocated tweets, representing more than a third of the total. This high proportion may reflect both the prevalence of Twitter usage in America, with the United States being the country with the most Twitter users (63), this data makes sense. Additionally, it is evident that there is interest and concern about this issue, and that in this country 1 out of every 5 deaths is a consequence of smoking tobacco (64).

The second continent with the highest number of tweets is Europe. Among the posts, a higher frequency of content related to frequent tobacco consumption is observed, which may indicate a concern for tobacco consumption patterns in the region. This result may be due to the fact that, according to the WHO global report, Europe is the second continent with the highest prevalence of smokers (65).

However, it is noteworthy that the African continent has the lowest percentage of tweets regarding the harm of tobacco use to health (41.6%), when compared to the rest of the continents. For example, in America, 53.1% of tweets mention the harms of tobacco to health; in Europe, 60.6%; in Asia, 74.2%; and in Oceania, 52.5%. This could be due to greater laxity in tobacco control policies (66, 67), with lower taxes and a stronger presence of the tobacco industry in this region (68).

Overall, these findings highlight the importance of considering geographical and cultural differences when designing strategies for tobacco prevention and control on a global scale.

Public health surveillance of behaviors, opinions, and attitudes on relevant health topics on social media has proven to be of great value for healthcare professionals. It allows us to focus on what our users truly think and thus develop more appropriate approaches to their needs and create efficient interventions to prevent future health issues (21).

## Limitations

This study has some limitations. Firstly, the social, economic, and demographic characteristics of Twitter users do not fully reflect society as a whole. Secondly, the design of the codebook and the analysis of tweets involve some subjectivity, as is common in qualitative studies. However, this methodology is consistent with previous medical research studies using Twitter. Additionally, to address this issue, our study included several countermeasures, such as an initial review, codebook design, and agreement among coders. Third, another limitation to note is that the use of the keyword tobacco probably limited the sample of tweets, since the people who use the products probably use the name of the product or hashtags such as vaping, juuling or smoking. So, because of the nature of the word tobacco it is likely that the sample was more of health professionals or other people who use the more formal term “tobacco” and it probably increased the likelihood that the tweets were about the harms of tobacco. Finally, it should be noted that the least represented continent has been Africa, probably because we have only collected tweets published in Spanish or English. In future work, consideration should be given to including languages that are more widely spoken on this continent.

## Conclusions

In conclusion, our study sheds light on several important aspects of tobacco discourse on Twitter and its implications for public health. Firstly, we found a notable emphasis on tweets discussing the harmful effects of tobacco, indicating a positive trend toward increased awareness among users.

Furthermore, our analysis reveals unexpected patterns of engagement, with tweets discussing both the benefits and harms of smoking garnering similar levels of interaction. This suggests the presence of influential accounts promoting tobacco-related content, potentially aligned with industry interests. The substantial presence of healthcare professionals on Twitter presents an opportunity for disseminating accurate information and preventive measures.

Geographically, America emerges as the predominant region for tobacco-related discourse on Twitter, followed by Europe. These findings underscore the need to tailor tobacco prevention strategies to regional differences and cultural contexts.

Overall, our study underscores the value of social media surveillance for understanding public attitudes toward tobacco and informing targeted interventions. By leveraging platforms like Twitter, healthcare professionals and institutions can amplify their efforts in combating tobacco use and promoting public health.

As future lines of research, we aim to include other keywords, such as vaping or electronic cigarettes, which would allow us to analyze discourse on Twitter regarding new forms of tobacco products. Additionally, we believe it would be interesting to expand the search to more languages to have greater representation in other geographical areas, which were minority in our study.

## Data availability statement

The raw data supporting the conclusions of this article will be made available by the authors, without undue reservation.

## Ethics statement

The studies involving humans were approved by Comité de Ética de la Investigación y de Experimentación Animal de la Universidad de Alcalá. The studies were conducted in accordance with the local legislation and institutional requirements. Written informed consent for participation was not required from the participants or the participants' legal guardians/next of kin in accordance with the national legislation and institutional requirements.

## Author contributions

CC-T: Conceptualization, Investigation, Methodology, Project administration, Writing – original draft. CF-L: Formal analysis, Methodology, Writing – review & editing. FL-A: Data curation, Formal analysis, Software, Writing – review & editing. RM-R: Visualization, Writing – review & editing. MO: Visualization, Writing – review & editing. FM: Visualization, Writing – review & editing. MA-M: Supervision, Writing – review & editing. JQ: Visualization, Writing – review & editing. MAA-M: Project administration, Supervision, Writing – review & editing.
